# Elevated visual dependency in young adults after chemotherapy in childhood

**DOI:** 10.1371/journal.pone.0193075

**Published:** 2018-02-21

**Authors:** Einar-Jón Einarsson, Mitesh Patel, Hannes Petersen, Thomas Wiebe, Per-Anders Fransson, Måns Magnusson, Christian Moëll

**Affiliations:** 1 Department of Clinical Sciences, Lund University, Lund, Sweden; 2 Faculty of Medicine, University of Iceland, Reykjavik, Iceland; 3 Division of Brain Sciences, Imperial College London, London, United Kingdom; 4 Department of Otorhinolaryngology, Landspitali University Hospital, Reykjavik, Iceland; 5 Department of Pediatrics, Skåne University Hospital, Lund, Sweden; 6 Department of Otorhinolaryngology, Skåne University Hospital, Lund, Sweden; National Cancer Institute, UNITED STATES

## Abstract

Chemotherapy in childhood can result in long-term neurophysiological side-effects, which could extend to visual processing, specifically the degree to which a person relies on vision to determine vertical and horizontal (visual dependency). We investigated whether adults treated with chemotherapy in childhood experience elevated visual dependency compared to controls and whether any difference is associated with the age at which subjects were treated. Visual dependency was measured in 23 subjects (mean age 25.3 years) treated in childhood with chemotherapy (CTS) for malignant, solid, non-CNS tumors. We also stratified CTS into two groups: those treated before 12 years of age and those treated from 12 years of age and older. Results were compared to 25 healthy, age-matched controls. The subjective visual horizontal (SVH) and vertical (SVV) orientations was recorded by having subjects position an illuminated rod to their perceived horizontal and vertical with and without a surrounding frame tilted clockwise and counter-clockwise 20° from vertical. There was no significant difference in rod accuracy between any CTS groups and controls without a frame. However, when assessing visual dependency using a frame, CTS in general (p = 0.006) and especially CTS treated before 12 years of age (p = 0.001) tilted the rod significantly further in the direction of the frame compared to controls. Our findings suggest that chemotherapy treatment before 12 years of age is associated with elevated visual dependency compared to controls, implying a visual bias during spatial activities. Clinicians should be aware of symptoms such as visual vertigo in adults treated with chemotherapy in childhood.

## Introduction

The number of childhood cancer survivors has dramatically increased with the invention of chemotherapy and testament to this is the increase in survival from osteosarcoma in childhood; from 21% between 1933 and 1959 before chemotherapy [1] to 75.5% in 2010 [2]. However, chemotherapeutic agents can cause long-term neurophysiological side-effects [3–6] including general impairment of brain function [7]. Recent findings also suggest that chemotherapeutic agents can alter the structure of the cortex [8, 9], basal ganglia and hippocampus [10] and thus, neurophysiological changes could extend to the spatial systems [11]. These impairments appear to be related to chemotherapeutic agents rather than the position of the cancerous tissue, since the impairments occur homogenously in spite of the fact that patient tumor locations occur heterogeneously [3, 11, 12].

Spatial orientation depends on the integration of visual, vestibular and somatosensory cues by the central nervous system (CNS) [13]. However, the weighting of each sensory system varies across the population [14]. An example of this variation is the degree to which a person relies on vision to make judgments about spatial position, termed visual dependency [15, 16]. The first study of visual dependency was conducted by Witkin and Asch, who reported that when participants were presented with tilted visual cues with respect to gravitational vertical, about half incorrectly perceived themselves as being tilted in the direction of the visual “frame” [16]. This effect has been replicated with the “Rod and Frame” test, in which a subject sits in a completely dark room, except for a dimly lit rod. The subject is then asked to position the rod to their perceived horizontal (subjective visual horizontal, SVH) and vertical (subjective visual vertical, SVV) orientations (a spatial alignment estimate) in isolation “no-frame” and in the presence of a static tilted frame [17, 18]. The difference in rod position (in degrees) between frame and no-frame tests is termed visual dependency, i.e., the position difference remaining when the rod position recorded with the no-frame test is subtracted from the position recorded with a tilted frame [15, 19]. Individuals who rely mainly on visual references (visually dependent) are biased by their visual perception of the frame’s orientation whereas individuals who use other sensory cues such as vestibular and somatosensory receptors (visually independent), position the rod to the true gravitational horizontal or vertical [20, 21].

The influence of peripheral and central nervous disorders on visual dependency has been explored with the Rod and Frame test. Although the biological basis of visual dependency is unclear, Parkinsonian patients [22], vestibular impaired patients [18, 23] and stroke sufferers [24] exhibit higher levels of visual dependency compared to age-matched controls. Visual dependency is also elevated in elderly fallers compared to non-fallers [25] and in subjects under the influence of alcohol intoxication [18]. Given these findings, a reasonable assumption is that visual dependency is a useful clinical measure.

Interestingly, the side effects of chemotherapy in childhood can include visual distortions, dizziness, poor attention and headaches in adulthood [11, 26], which might be associated with elevated visual dependency. The aim of this study was therefore to investigate visual dependency in adults treated in childhood for cancer with chemotherapy compared to age-matched controls. As the peripheral and central nervous systems involved in postural control are still in development before 12 years of age [27], a second aim was to investigate whether the age at which subjects were treated influences visual dependency. Intriguingly, Einarsson and colleagues [11] recently found that subjects treated before 12 years of age experienced greater vertigo symptoms and poorer control of eye movement compared to subjects treated from 12 years of age. We therefore also compared visual dependency in subjects treated before 12 years of age to controls, subjects treated from 12 years of age to controls and subjects treated before 12 years of age to subjects treated from 12 years of age. We hypothesize that chemotherapy treatment in childhood elevates visual dependency compared to controls, but particularly in subjects treated before 12 years of age.

The SVV and SVH are measures both assumed to probe the underlying perception of gravity. However, when the body is roll tilted, these two measures evoke different patterns of errors with SVV generally becoming biased towards the roll tilted body (denoted A-effect) and SVH remaining accurate or becoming biased away from the tilted body (denoted E-effect) [28, 29]. This effect may be due to a difference in how sensory information is transduced by the head (vestibular system) and by the body (somatosensation, somatic graviception [30]). Intriguingly, Bronstein et al. [31] reported that vestibular nuclear lesions lead to a distortion of SVV, but not SVH. Also, a counter-clockwise frame tilt has been found to cause a larger rod position error than a clockwise frame tilt [18]. Both effects (SVV/SVH orientation and frame tilt direction) were explored in this study. This study is part of a series in which adults treated for cancer with chemotherapy in childhood or adolescence were screened for hearing and sensorimotor impairments [11, 12, 32, 33].

## Materials and methods

### Ethics statement

As described before [11, 12, 32, 33], experiments were performed in accordance with the Helsinki declaration and approved by the Scientific Ethical Committee at Lund University, Sweden (number LU964-03) and the Data Protection Authority, Sweden (number LU-P6103). A non-obstat statement was obtained from the Scientific Ethical Committee, stating that no additional ethical approvals were required to perform the investigations for the follow-up of this population. All participants or their guardians provided written informed consent before testing.

### Subjects

As described in detail previously [11, 12, 32, 33], forty-eight subjects, 23 chemotherapy treated subjects (CTS) and 25 healthy controls were recruited. The CTS were recruited from all adults surviving childhood cancer in the county of Skåne, Sweden, between 1980 and 2000. From approximately 750 adult survivors, 23 fulfilled the strict inclusion criteria: cancer diagnosed before the age of 18; treated for a solid malignant non-CNS tumor with chemotherapy agents and the treatment completed >5 years before this study. Subjects who had received cranial radiotherapy or surgery, which might affect the CNS, were excluded from participation. The most common reason for exclusion was that the individual had cancer in locations inside or in close proximity to CNS structures or that other kinds of treatments than chemotherapy might have damaged CNS structures, e.g., surgery or radiation. Another common reason for exclusion was that the cancer was not restricted to a local area, i.e., it did not fulfill the solid tumor criteria. Subjects fulfilling criteria were contacted by a clinical administrator and offered to participate in the study, which they all did. The investigations were performed as part of a clinical follow-up of the patient population.

The 23 CTS were 11 females and 12 males of mean age 25.3 years (SD 6.7). Mean age at diagnosis and treatment was 10.2 years (SD 5.1). The tests in this study were performed mean 15.1 years (SD 5.6) after the end of chemotherapy treatment. The treatment details for the CTS population, as described in detail previously [11], are presented in Table 1. To determine whether the age at which subjects were treated influenced visual dependency, we stratified CTS into two subgroups; CTS_Young: 14 subjects treated before 12 years of age (8 women, mean age 23.6 years (SD 7.0)) with mean age at time of treatment of 7.0 years (SD 3.5); and CTS_Old: 9 subjects treated from 12 years of age and older (3 women, mean age 28.0 years (SD 5.4)) with mean age at time of treatment of 15.3 years (SD 1.9). Mann-Whitney between-group analyses showed that the frequency and dosage of chemotherapy did not significantly differ between CTS_Young and CTS_Old (p>0.05). The age threshold of 12 years was selected as the systems involved in postural control are still in development before 12 years of age [27]. Furthermore, this division has revealed age-associated effects in the same cohort previously [11, 12]. All subjects included in the study, both controls and CTS, had normal or corrected to normal visual acuity using glasses or contact lenses.

**Table 1:** Subject characteristics, diagnosis and chemotherapy details.

Subject	Gender	Age at treatment (years)	Age at assessment (years)	Duration of treatment (weeks)	Diagnosis	Chemotherapy treatment agents ^1^
1	Female	0.1	23.4	10	Sacrococcygeal teratoma	Ble, Cis, Eto
2	Female	2.5	15.9	20	Hepatoblastoma	Adr, Cis
3	Female	2.5	17.7	14	Embryonal teratoma	Ble, Cis, Eto
4	Male	2.9	16.4	35	Ewing sarcoma	Act, Adr, Eto, Ifo, Vin
5	Female	6.1	17.5	27	Osteosarcoma	Adr, Cis, Met
6	Female	8.4	15.5	46	Osteosarcoma	Adr, Cis, Ifo, Met
7	Female	8.6	30.0	62	Ewing sarcoma	Act, Adr, Ble, Cyc, Met, Vin
8	Male	8.7	27.7	58	Ewing sarcoma	Act, Adr, Ble, Cyc, Met, Vin
9	Male	8.9	21.4	12	Neuroblastoma	Car, Cis, Cyc, Eto, Mel, Vin
10	Female	9.1	18.5	8	Immature teratoma	Ble, Cis, Eto
11	Male	9.6	30.3	58	Ewing sarcoma	Act, Adr, Ble, Cyc, Met, Vin
12	Male	9.9	27.6	39	Osteosarcoma	Act, Adr, Ble, Cis, Cyc, Met
13	Female	10.3	35.8	58	Immature teratoma	Act, Adr, Cyc, Vin
14	Male	10.7	33.1	49	Ewing sarcoma	Act, Adr, Ble, Cis, Cyc, Met, Vin
15	Female	12.1	18.4	39	Ewing sarcoma	Act, Adr, Cyc, Eto, Ifo, Vin
16	Female	12.6	27.4	25	Osteosarcoma	Adr, Cis, Met
17	Female	14.3	33.9	29	Osteosarcoma	Act, Adr, Ble, Cis, Cyc, Met
18	Male	15.5	35.4	65	Ewing sarcoma	Act, Adr, Ble, Cyc, Met, Vin
19	Male	15.7	24.0	31	Ewing sarcoma	Adr, Cis, Ifo, Vin
20	Male	16.5	27.8	9	Immature teratoma	Ble, Cis, Eto
21	Male	16.8	23.7	76	Ewing sarcoma	Act, Adr, Cis, Cyc, Eto, Ifo, Vin
22	Male	16.9	30.9	27	Osteosarcoma	Adr, Cis, Met
23	Male	17.0	30.4	23	Osteosarcoma	Adr, Cis, Eto, Ifo, Met

^1^ Act: Actinomycin-D; Adr: Adriamycin; Ble: Bleomycin; Car: Carboplatin; Cis: Cisplatin; Cyc: Cyclophosphamide; Eto: Etoposide; Ifo: Ifosfamide; Mel: Melphalan; Met: Methotrexate; Vin: Vincristine.

### Test procedure

**Fig 1:**
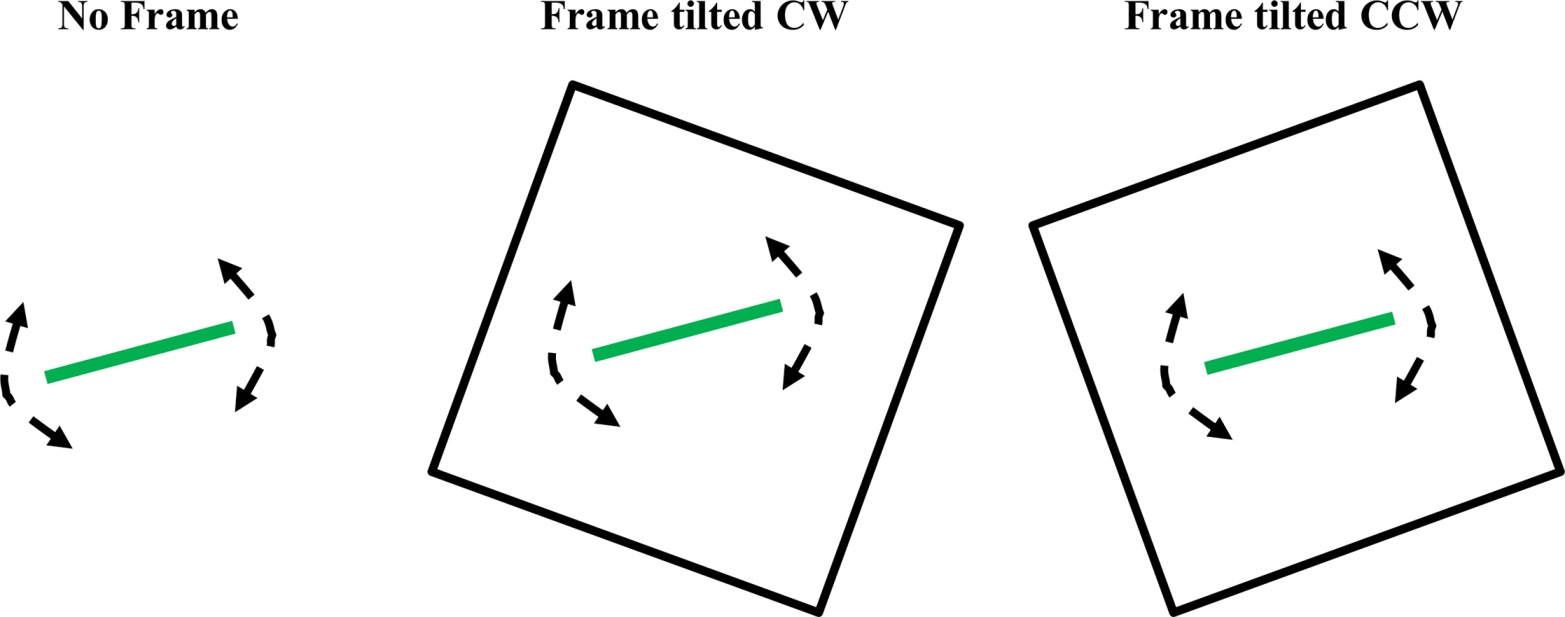
Illustration of no frame and rod & frame tests. The subjects were instructed to position a green rod four times each in perfect horizontal or vertical directions. The rod was rotated around its centered axis, whereas the position and rotation of the frames were fixed during the assessments. The brightness of the rod and the frames were fixed. Before testing commenced it was ensured that the brightness was sufficient to allow the test subject to clearly detect the rod and frames.

The term “visual dependency” in this paper is defined as the perception of spatial orientation from a static visual reference. The subjects sat upright in a completely dark room, with their head immobilized using adjustable neck straps. Tests of SVV and SVH are commonly performed sitting in order to produce a test situation where the sensory information is lower from proprioception and from the mechanoreceptors in the feet. Thus, the subjects should experience a situation where they have to rely more than usual on information from vision and the vestibular systems when determining the spatial orientation [18]. An illuminated rod (15 cm x 0.5 cm), which could be rotated about its mid-point, was projected onto a wall 1.5 m in front of participants, see Fig 1. The subjects were instructed to rotate the rod to the perceived horizontal (SVH) or vertical (SVV) using a remote control with three buttons; two buttons for rotating the rod in clockwise (CW) and counter-clockwise (CCW) directions, and a third button to indicate when the rod had the orientation requested. Briefly pressing the CW or CCW buttons produced small angle adjustments with a resolution of 0.03 degrees. Continuously pressing the CW or CCW buttons produced a slow rotation at 0.6 degree/s of the rod initially, whereas holding the buttons down for longer than 5 seconds produced a fast rotation at 14 degree/s.

Before the start of each positioning task, the rod was randomly positioned 20–40 degrees CW or CCW from true horizontal (SVH task) or vertical (SVV task). After the subject confirmed having completed adjusting the rod position so it was now perceived to be perfectly aligned with the requested either vertical or horizontal orientation, the accuracy of the SVH and SVV was calculated as the difference in final rod position (in degrees) compared to the true vertical or horizontal. A CCW tilt from true vertical or horizontal produced a negative value and a CW tilt produced a positive value.

### No frame tests

The illuminated rod was projected onto a wall in an otherwise completely dark room and without visual references. Each subject was asked to rotate the rod to the requested position eight times in total, four times to perceived horizontal orientation and four times to perceived vertical orientation, in a randomized sequence switching between horizontal and vertical positioning tasks. After the test was completed, mean signed rod accuracy values were calculated for the 4 positioning tasks performed in each vertical and horizontal orientations, the mean values denoted as SVV_NoFrame_ and SVH_NoFrame_ respectively. Moreover, to determine whether the rod accuracy values were of similar sizes irrespective of whether the orientation errors were CW and CCW, the absolute SVH_NoFrame_ and SVV_NoFrame_ values were calculated and analyzed.

### With frame tests (visual dependency)

The illuminated rod was projected onto a wall in an otherwise completely dark room, though this time the rod was surrounded by an illuminated frame (100 cm x 100 cm), tilted either 20° counter-clockwise or 20° clockwise, see Fig 1. Like the no-frame test, each subject was asked to rotate the rod to the requested position eight times in total, four times to perceived horizontal orientation and four times to perceived vertical orientation, in a randomized sequence switching between horizontal and vertical positioning tasks. This procedure was repeated twice, once with the frame tilted CW and once with the frame tilted CCW, with the order of the two different frame tilts randomized between subjects. After the test was completed, mean signed rod accuracy values were calculated for the four SVH positioning tasks and the four SVV positioning tasks: the mean values denoted as SVH_Frame_ and SVV_Frame_.

Visual dependency is defined as the difference in mean rod accuracy (in degrees) between tests with a tilted frame and without a frame [15, 18, 19]. Hence, we subtracted the respective SVH_NoFrame_ and SVV_NoFrame_ values recorded without a frame from the SVH_Frame_ and SVV_Frame_ values recorded with a CW or CCW leaning frame, subsequently producing visual dependency values for horizontal and vertical orientations;

$$SVH_{VD}\left(CW\right)={SVH}_{Frame}\left(CW\right)-{SVH}_{NoFrame}$$ (1)

$$SVV_{VD}\left(CW\right)={SVV}_{Frame}\left(CW\right)-{SVV}_{NoFrame}$$ (2)

$$SVH_{VD}\left(CCW\right)={SVH}_{Frame}\left(CCW\right)-{SVH}_{NoFrame}$$ (3)

$$SVV_{VD}\left(CCW\right)={SVV}_{Frame}\left(CCW\right)-{SVV}_{NoFrame}$$ (4)

Moreover, to determine whether the rod accuracy values were of similar sizes irrespective of whether the orientation errors were in CW and CCW directions the absolute SVH_VDFrame_ and SVV_VDFrame_ values were calculated and analyzed.

### Statistical analysis

The signed and absolute SVH and SVV accuracy values were analyzed using repeated measures General Linear Model (GLM) ANOVA for four group constellations; CTS vs. Controls, CTS_Young vs. Controls, CTS_Old vs. Controls, CTS_Young vs. CTS_Old. The main factors when analyzing data from tests without a frame were: “Chemotherapy”: (e.g., CTS vs. Controls; 1d.f); and “Orientation“: (SVH vs. SVV; 1d.f). The main factors when analyzing data from tests with a frame (visual dependency) were: “Chemotherapy”: (e.g., CTS vs. Controls; 1d.f); “Orientation“: (SVH vs. SVV; 1d.f) and “Frame”: (CW vs CCW tilt; 1d.f).

**Fig 2:**
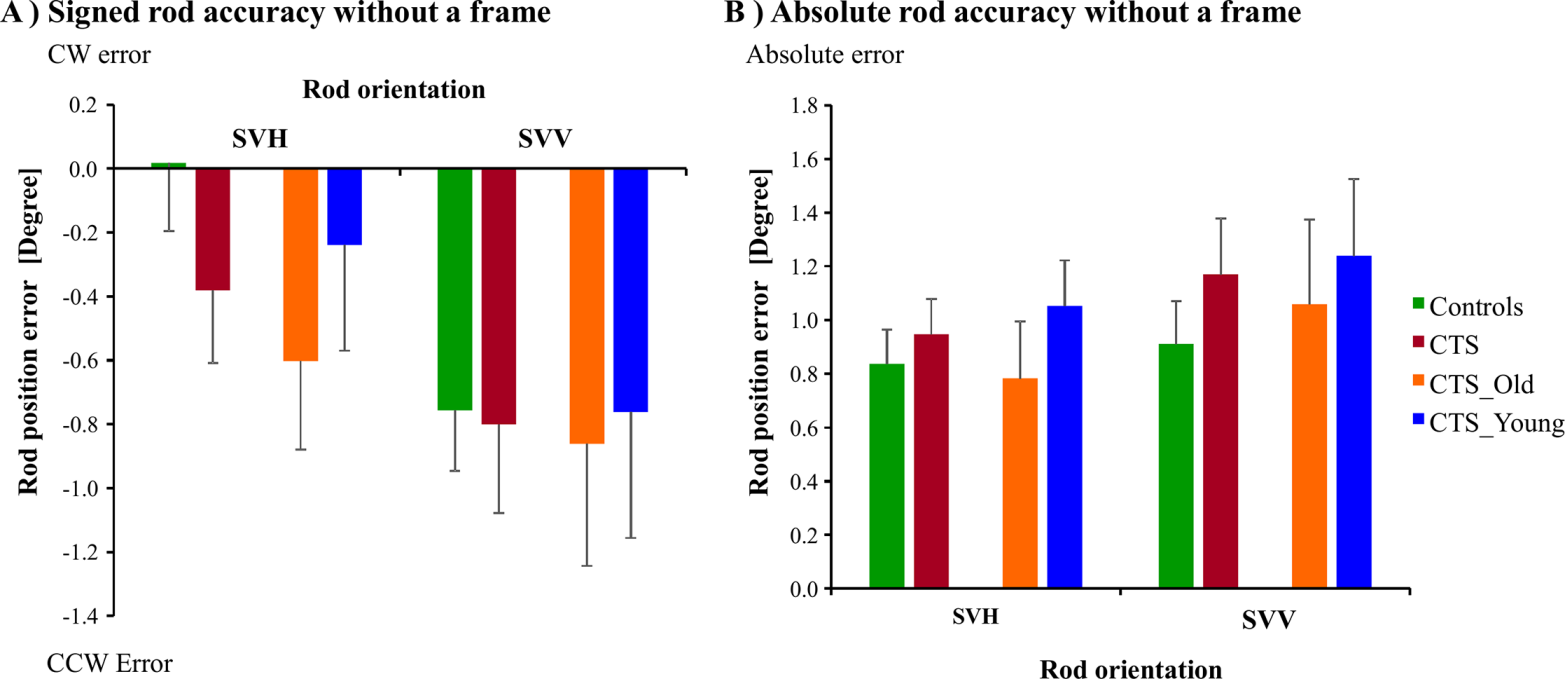
**(A) Signed and (B) Absolute rod inaccuracy (mean and SEM) when the tests were performed without a frame around the rod.** Positive values illustrate a CW inaccuracy and negative values illustrate a CCW inaccuracy from correct spatial orientation.

The Mann-Whitney U (Exact sig. 2-tailed) test was used for a separate between-group post hoc comparisons on Group, Orientation and Frame subgroups, as both the between-subject factor and the within-subjects variables were evidenced in the repeated measured GLM ANOVA to have a significant effect, see Fig 2. In all GLM ANOVA tests, p< 0.05 was considered significant whereas in the Mann-Whitney comparisons p<0.025 was considered significant following Bonferroni correction [34]. Non-parametric statistical tests were used in the post hoc evaluations, as some datasets were not normally distributed following Shapiro-Wilk testing. The repeated measures GLM ANOVA analysis was used after ensuring that all model residuals had normal or approximate normal distribution. A sample size analysis, using the statistical package G-power™ and with parameters set to; effect size = 0.7 and p = 0.05 2-tailed; showed that the study would require about n = 10 subjects per group to reach a power value of 0.8. The statistical analyses were performed with SPSS version 24 and the power analysis was performed GPower 3.

## Results

### No frame test

#### Signed values

When evaluating the signed values, we found no significant difference between any groups in rod accuracy without a frame, see Table 2. However, the rod accuracy was significantly poorer in SVV orientation than in SVH orientation in all groups (CTS vs. Controls, (p<0.001); CTS_Young vs. Controls, (p = 0.003); CTS_Old vs. Controls, (p<0.001); CTS_Young vs. CTS_Old, (p = 0.032). There was no evidence of interactions between Chemotherapy and Orientation for any group. The post-hoc tests revealed no significant group differences in rod accuracy without a frame, see Fig 2A.

**Table 2:** Effects of chemotherapy and orientation on rod accuracy.

		Chemotherapy ^1^	Orientation	Chemotherapy x Orientation
CTS vs controls	Signed	0.448 [0.6]	**< 0.001 [18.5]**	0.208 [1.6]
	Absolute	0.310 [1.1]	0.283 [1.2]	0.588 [0.3]
CTS_Young vs controls	Signed	0.251 [1.4]	**0.003 [10.0]**	0.103 [2.8]
	Absolute	0.126 [2.5]	0.436 [0.6]	0.765 [0.1]
CTS_Old vs controls	Signed	0.954 [0.0]	**<0.001 [15.7]**	0.849 [0.0]
	Absolute	0.999 [0.0]	0.322 [1.0]	0.539 [0.4]
CTS_Young vs CTS_Old	Signed	0.414 [0.7]	**0.032 [5.3]**	0.268 [1.3]
	Absolute	0.318 [1.0]	0.267 [1.3]	0.730 [0.1]

Repeated measures GLM ANOVA analysis of how the rod accuracy was affected by main factors “Chemotherapy” and SVH/SVV “Orientation” alone and by the main factor interaction denoted as “Chemotherapy x Orientation”. The notation “<0.001” means that the p-value is smaller than 0.001. F-values are presented in the squared parenthesis.

^1^ In the CTS_Young vs CTS_Old GLM ANOVA evaluation the main factor “Chemotherapy” represents the effect of receiving chemotherapy below 12 years of age (CTS_Young) vs from 12 years of age and older (CTS_Old).

#### Absolute values

For the absolute values, i.e., the absolute sizes of rod accuracy, another picture emerged. There were no significant differences between groups in rod accuracy, see Table 2. There was no significant difference between SVV and SVH orientations, and no significant interactions between Chemotherapy and Orientation. The post-hoc tests revealed no significant differences between groups in absolute rod accuracy without a frame, see Fig 2B.

### With frame tests (visual dependency)

#### Signed values

When evaluating the signed values, we found no significant difference between any groups in rod accuracy, see Table 3. Moreover, we found no significant difference between SVV and SVH orientation in any group. However, CCW frame tilts produced a significantly different rod inaccuracy than CW frame tilts. The CCW frame tilts produced a CCW rod inaccuracy whereas CW frame tilts produced a CW rod inaccuracy (CTS vs. Controls, (p<0.001); CTS_Young vs. Controls, (p<0.001); CTS_Old vs. Controls, (p<0.001); CTS_Young vs. CTS_Old, (p<0.001).

**Table 3:** Effects of chemotherapy, orientation and frame tilt on rod accuracy.

		Chemo	Orientation	Frame	Chemo x Orientation	Chemo x Frame	Orientation x Frame	Chemo x Orientationx Frame
CTS vs controls	Signed	0.367 [0.8]	0.701 [0.1]	**< 0.001 [80.3]**	0.546 [0.4]	**0.003 [10.2]**	**<0.001 [22.1]**	0.217 [1.6]
	Absolute	**0.006 [8.4]**	**<0.001 [17.9]**	**0.001 [12.0]**	0.088 [3.0]	0.153 [2.1]	0.780 [0.1]	0.499 [0.5]
CTS_Young vs controls	Signed	0.183 [1.8]	0.927 [0.0]	**< 0.001 [74.6]**	0.488 [0.5]	**< 0.001 [13.6]**	**0.001 [11.8]**	0.855 [0.0]
	Absolute	**0.001 [12.6]**	**0.003 [9.7]**	**< 0.001 [14.4]**	0.417 [0.7]	0.064 [3.6]	0.882 [0.0]	0.360 [0.9]
CTS_Old vs controls	Signed	0.958 [0.0]	0.506 [0.5]	**< 0.001 [35.4]**	0.875 [0.0]	0.229 [1.5]	**< 0.001 [30.1]**	**0.018 [6.3]**
	Absolute	0.365 [0.8]	**< 0.001 [20.1]**	**0.035 [4.9]**	**0.019 [6.2]**	0.639 [0.2]	0.390 [0.8]	0.970 [0.0]
CTS_Young vs CTS_Old	Signed	0.460 [0.6]	0.962 [0.0]	**< 0.001 [61.1]**	0.657 [0.2]	0.072 [3.6]	**0.001 [13.4]**	0.145 [2.3]
	Absolute	0.068 [3.7]	**0.001 [14.2]**	**0.025 [5.9]**	0.214 [1.6]	0.442 [0.6]	0.931 [0.0]	0.465 [0.6]

Repeated measures GLM ANOVA analysis of how the rod accuracy was affected by main factors “Chemotherapy” (denoted Chemo in the table), SVH/SVV “Orientation” and CW/CCW “Frame” tilt direction alone and by their main factor interactions.

Moreover, the significant interaction between Chemotherapy vs Frame reveal that CTS (p = 0.003) and CTS_Young (p<0.001) had significantly larger rod inaccuracy than controls during CCW frame tilts than during CW frame tilts. Furthermore, the Orientation vs. Frame interaction shows that the rod inaccuracy difference between SVH and SVV orientation was significantly larger for CW frame tilts than for CCW frame tilts in all groups (CTS vs. Controls, (p<0.001); CTS_Young vs. Controls, (p = 0.001); CTS_Old vs. Controls, (p<0.001); CTS_Young vs. CTS_Old, (p = 0.001). Finally, the Chemotherapy vs. Orientation vs. Frame interaction reveals that chemotherapy group changed the level of difference in rod inaccuracy between SVH and SVV orientation which varied for CW and CCW frame tilts. The SVH vs. SVV difference were large in the CTS_Old with CW frame tilt whereas the SVH vs. SVV difference were large in the controls with CCW frame tilts (p = 0.018).

**Fig 3:**
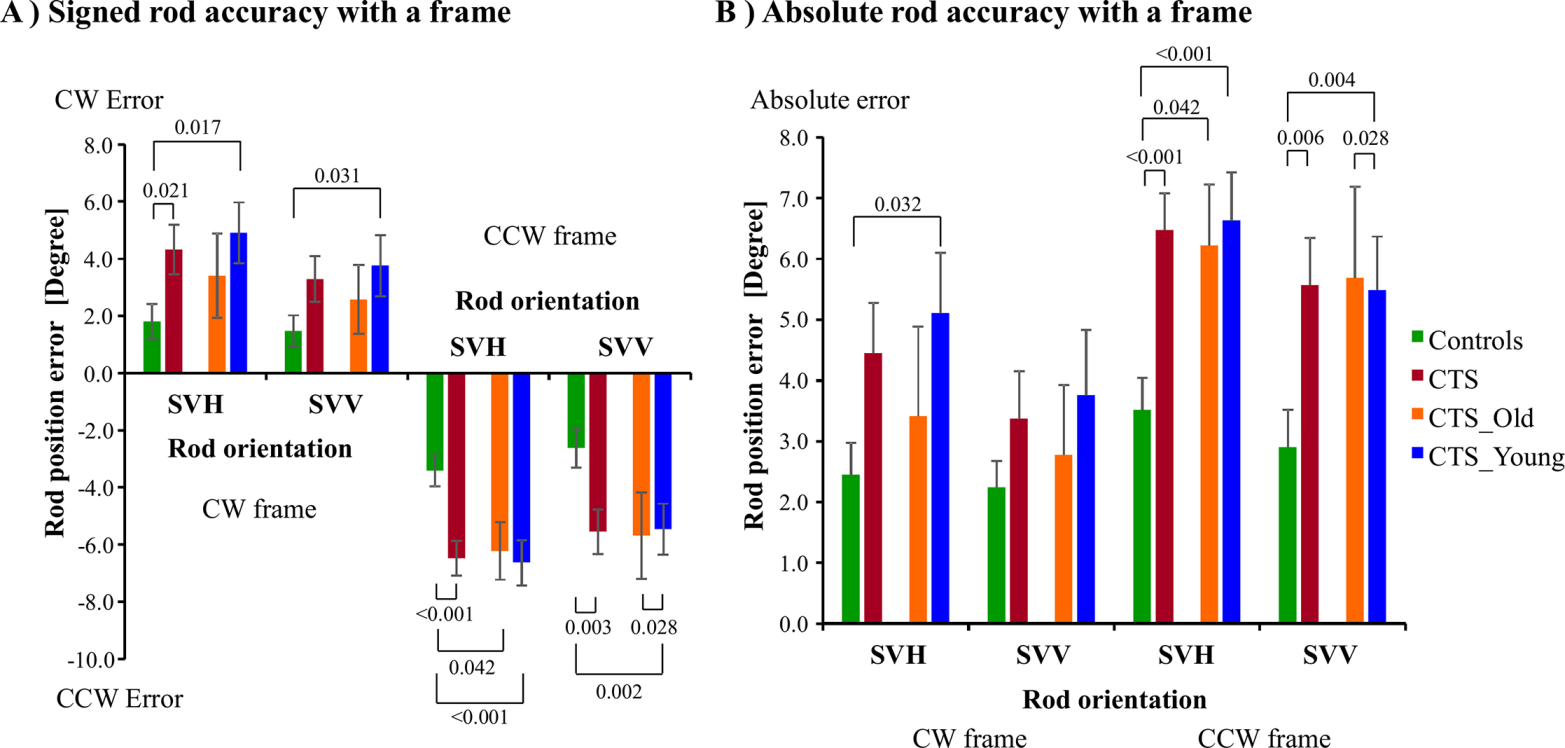
**(A) Signed and (B) Absolute rod inaccuracy (mean and SEM) when the tests were performed with a frame around the rod, the frame tilted either CW or CCW.** The Bonferroni corrected significance level is in the between-groups analyses set to p<0.025, though for consistency reasons p-values to the level of 0.05 are also presented in the figure.

The post-hoc tests revealed that for CW frame tilts, rod inaccuracy was significantly larger in CTS (p = 0.021) and CTS_Young (p = 0.017) than controls in SVH orientation. Moreover, for CCW frame tilts, rod inaccuracy was significantly larger in CTS (p<0.001) and CTS_Young (p<0.001) than controls in SVH orientation, and the rod inaccuracy was significantly larger in CTS (p = 0.003) and CTS_Young (p = 0.002) than controls in SVV orientation, see Fig 3A.

#### Absolute values

When evaluating the visual dependency using frames, we found significantly larger absolute rod inaccuracies for CTS (p = 0.006) and CTS_Young (p = 0.001) than controls, see Table 3. Moreover, the rod inaccuracy was significantly larger in SVH orientation than in SVV orientation in all groups (CTS vs. Controls, (p<0.001); CTS_Young vs. Controls, (p = 0.003); CTS_Old vs. Controls, (p<0.001); CTS_Young vs. CTS_Old, (p = 0.001). Furthermore, CCW frame tilts produce significantly larger rod inaccuracy than CW frame tilts in all groups (CTS vs. Controls, (p = 0.001); CTS_Young vs. Controls, (p<0.001); CTS_Old vs. Controls, (p = 0.035); CTS_Young vs. CTS_Old, (p = 0.025).

The Chemotherapy vs. Orientation interaction reveals that the rod inaccuracy difference between SVH and SVV orientation was significantly larger in CTS_Old than in Controls (p = 0.019).

The post-hoc tests revealed that for CW frame tilts, the absolute rod inaccuracy was not significantly different between groups. However, for CCW frame tilts, the rod inaccuracy was significantly larger in CTS (p<0.001) and CTS_Young (p<0.001) than for controls in SVH orientation, and the rod inaccuracy was significantly larger in CTS (p = 0.006) and CTS_Young (p = 0.004) than for controls in SVV orientation, see Fig 3B.

## Discussion

Higher levels of visual dependency have been found in several patient groups including elderly fallers [25], Parkinsonian patients [22], vestibular impaired subjects [18, 23] and stroke patients [24]. Elevated visual dependency also increases the risk of visually-induced vertigo, i.e., dizziness, light-headedness, unsteadiness and disorientation in visually chaotic environments [35]. Intriguingly, symptoms of visually-induced vertigo have been found in adults treated with chemotherapy in previous studies [11, 26]. Here, we show for the first time that adults treated with chemotherapy before 12 years of age are commonly more visually dependent than controls. This finding may originate from several causes: One possibility could be a higher weighting of visual cues following neurotoxic or ototoxic damage to the other sensory systems from chemotherapy in childhood. Another possibility is that the “re-weighting” of sensory signals is part of the recovery process and reduces symptoms [19] through a higher weighting of visual cues. A third possibility could be that chemotherapy causes refractive errors influencing the visual perspective. Visual system abnormalities, such as slower pursuits and weaker saccades, are evident in this population [11], but the mechanism by which these voluntary physiological outputs occur are not the same as the cognitive mechanisms which determine our sense of spatial orientation. Of note, when only a rod was visible, the CTS could handle the task of positioning this rod horizontally and vertically equally well as the controls.

Higher visual dependency has been found in patients with peripheral or central vestibular disorders who experience visual vertigo [36]. In the ‘rod and disk’ examination, Guerraz and colleagues (2001) found that patients with visual vertigo perceived vertical to be about 6.5 degrees from gravitational vertical in the presence of background ‘disk’ motion. Strikingly, we observed that the CTS had a similar sized mean deviation of about 6.6 degrees in the face of counter-clockwise frame tilts. This counter-clockwise effect could be explained by the ‘A-effect’ which is associated with an inherent bias in sensing a body tilt [37]. Even in the absence of a frame, CTS deviated their signed spatial position estimate further counter-clockwise compared to controls, though not to a significant extent. When tilted frames were introduced, both controls and CTS produced position errors in the same direction as the CW and CCW tilted frames used (p<0.001) and the absolute position errors were tilted more by a CCW frame tilt than by a CW frame tilt (p≤0.035). Finally, the significant interaction between Chemotherapy vs Frame showed that CTS (p = 0.003) and CTS_Young (p<0.001) had significantly larger rod inaccuracy than controls during CCW frame tilts than during CW frame tilts. Hence, the spatial vision perception could in CTS easily be disrupted when surroundings do not align with gravitational vertical. It is unclear why CCW frame tilts both in CTS and controls produced larger spatial distortions than CW frame tilts. Noteworthy, many human functions have a side-dominance, e.g., right-handedness and the right eye visual perception preferences. However, a factor that might merit more attention is whether cultural factors might influence the visual perception, e.g., whether one is used to reading from right to left, and thus, is more used to scrutinize visual symbols from a certain perspective. None of the subjects in this study were of Arabic or Asian origin.

Unexpectedly, we also found differences in accuracy between the SVV and SVH tests. In the absence of the frame, subjects positioned the rod closer to the true horizontal than to the true vertical (p<0.001). However, with a frame, subjects positioned the rod closer to the true vertical than to the true horizontal (p<0.001). This said, the differences between SVV and SVH were less than 1 degree in size, thus, though the differences were markedly systematic the two measures are highly inter-correlated [38] in this study.

The age of subjects at the time of treatment might be an important factor in determining the level of morbidity following chemotherapy [39]. We have shown previously that subjects treated before 12 years of age experience poorer postural control, poorer oculomotor control and higher levels of light-headedness, visual disturbances and headaches compared to subjects treated from 12 years of age and older [11, 12]. Similarly, we found that subjects treated before 12 years of age experience higher visual dependency compared to controls (p = 0.001), whereas subjects treated from 12 years of age and older did not perform differently to controls (p = 0.365). That said, it is important not to regard age cutoffs too literally or dogmatically. Hence, several individual CTS_Young performed the tests almost as accurately as controls whereas some CTS_Old performed the tests as poorly as CTS_Young subjects did. The exact reason why age at treatment is of such importance is unclear, but it could reflect an increased vulnerability in the central nervous system to some chemotherapy agent when the CNS structures still are undergoing development at an earlier age.

Recent findings suggest that a higher number of chemotherapeutic agents can impair general brain function than previously believed [7]. Indeed, chemotherapeutic agents are associated with alterations of brain structure [8, 9] and significant neurotoxicity in different brain regions including the cortex, basal ganglia and hippocampus [10]. Although the study population was of insufficient size to determine which agents or dosages were responsible for elevated visual dependency, a review of the literature show that vincristine, cisplatin and methotrexate can damage the CNS and visual system [40–42], with effects appearing several years after treatment [43]. Furthermore, cisplatin is ototoxic, and the resulting damage to the vestibular system [44] could increase the weighting of visual cues for spatial orientation [17, 45]. Moreover, chemotherapeutic agents including cisplatin are more toxic to CNS progenitor cells and oligodendrocytes than cancer cell lines at clinical doses [46].

During childhood, critical neural processes occur, including proliferation of oligodendrocytes, redefinition and establishment of all neural connections and myelination of axons [9]. Damage to neural components from chemotherapy at this critical period may cause general long-term CNS impairment, possibly resulting in increased visual dependency. Hence, it is probably at this time, during development, that the damaging effects of chemotherapy are the greatest. As described by Dieterich “…damage to immature cell types, such as stem cells and progenitor cells, is likely to have a more profound impact on cellular plasticity and on the long-term outcome than isolated damage to more mature and differentiated cell types, which may be replenished from immature progenitor cells…” [3]. These complications from chemotherapy may be the result of direct neurotoxicity or indirect immune processes [47].

### Summary

Adults treated with chemotherapy before 12 years of age commonly experience elevated visual dependency compared to controls. From a clinical standpoint, clinicians should be aware of visually-induced vertigo and related symptoms in adults treated with chemotherapy in childhood. Individuals expressing these signs may benefit from visual desensitization exercises.
